# A deep learning-driven automated treatment planning framework for cervical cancer patients treated with volumetric modulated arc therapy

**DOI:** 10.1186/s13014-026-02842-9

**Published:** 2026-04-14

**Authors:** Boda Ning, Xiuyan Liang, Zhenguo Cui, Yingfa Li, Qi Liu, Shuaining Ma, Xiting Chen, Shanshan Yang, Yanling Bai, Deyang Yu

**Affiliations:** 1 Department of Radiation Physics, Harbin Medical University Cancer Hospital, Harbin, 150081 China; 2 Department of Gynecological Radiotherapy, Harbin Medical University Cancer Hospital, Harbin, 150081 China; 3 School of Biomedical Engineering, Capital Medical University, Beijing, 100069 China

**Keywords:** Auto-planning, Deep learning, Dose prediction, Treatment planning system script, Radiotherapy

## Abstract

**Background and purpose:**

The rapid and efficient generation of high-quality, dose-consistency volumetric modulated arc therapy (VMAT) plans remains challenging in radiotherapy. This study proposes a deep learning (DL) end-to-end (E2E) auto-planning framework and validate its practicality and feasibility for clinical implementation.

**Materials and methods:**

A total of 458 cervical cancer VMAT plans were enrolled and split into training, validation, and test cohorts. An E2E auto-planning framework with a two-stage cascaded DL network was developed: Stage 1 predicted coarse dose from CT and structure masks, and Stage 2 refined it using four beam-band priors and a composite loss. Dose-volume histogram (DVH) endpoints from refined predicted dose were converted into Monaco objectives via a scripting module for iterative optimization. Performance was evaluated with Dose, DVH, and snDVH scores, ablations, and comparisons with manual plans in terms of quality, clinical evaluation and deliverability.

**Results:**

The proposed DL method achieved the best performance, with Dose score, DVH score and snDVH score of 2.114 ± 0.218 Gy, 1.194 ± 0.295 Gy and 2.027 ± 0.586, respectively. Compared with manual plans, E2E auto-plans preserved target volume coverage while reducing all DVH metrics for bladder, rectum, small intestine, and spinal cord by 2% − 35% (all *p* < 0.05). The gamma passing rate of E2E auto-plans was higher than manual plans in the 3%/3 mm gamma criterion (98.1% vs. 97.9%).

**Conclusion:**

The proposed auto-planning framework demonstrated a high level of automation and clinical applicability, offering a reliable and promising tool to support radiotherapy workflows.

**Clinical trial number:**

Not applicable.

**Supplementary Information:**

The online version contains supplementary material available at 10.1186/s13014-026-02842-9.

## Introduction

Intensity-modulated radiotherapy (IMRT) and volumetric modulated arc therapy (VMAT) are widely used for cervical cancer (CC) because they enable highly conformal target coverage while improving sparing of surrounding organs at risk (OARs) through inverse planning and modulation [1–3]. In routine treatment, prescription and clinical priorities are determined by radiation oncologists, whereas plan generation relies on medical physicists to iteratively adjust optimization objectives and parameters until institutional and guideline-based criteria are satisfied [4]. However, both the quality and efficiency of manual planning largely depend on planner experience, and plan quality can vary across individual planners, posing a major challenge to workflow efficiency in busy clinics [5].

To improve efficiency and reduce variability, automated treatment planning (ATP) has been extensively studied [6]. Knowledge-based planning (KBP) is widely used to improve planning efficiency and consistency by modeling planning objectives to guide the treatment planning workflow, thereby minimizing planner intervention [7–9]. Dose-volume histogram (DVH)-based prediction is one established KBP approach, in which predicted DVH endpoints are used as planning goals; however, accuracy can be limited because the detailed space information of three-dimensional (3D) dose distribution is not explicitly modeled [10, 11]. In parallel, deep learning (DL)-based dose prediction has advanced rapidly, enabling fast generation of patient-specific 3D dose distributions from planning CT and structure masks after training on prior high-quality plans [12–19]. Nevertheless, even highly accurate predicted dose distributions are not inherently deliverable, and the gap between “dose prediction” and “clinically executable plans” remains a key barrier to achieve a closed-loop workflow.

Recent studies have begun to bridge this gap by converting DL-predicted dose distributions into deliverable IMRT or VMAT plans [20–22]. However, whether predicted doses can be translated robustly into treatment planning system (TPS) objectives that converge reliably to high-quality, deliverable plans requires further validation. This need is most evident in steep dose fall-off regions and at target-OAR interfaces, where anatomy-only prediction may not adequately reflect the directionality and gradient constraints imposed by inverse planning, limiting robust translation into stable TPS objectives and consistent plan quality. We therefore propose a two-stage cascaded DL framework that incorporates directional beam band priors and a DVH-oriented composite loss, and converts the refined dose into patient-specific, Monaco-executable objectives via in-house TPS scripting for automated inverse optimization. This study aimed to establish and validate an end-to-end (E2E) VMAT auto-planning workflow for CC and to assess dose prediction accuracy, plan quality, and deliverability.

## Materials and methods

### Patients and clinical plans

This retrospective study was approved by the Institutional Review Board of Institution A. A total of 458 CC patients treated with full-arc VMAT between 2023 and 2025 were enrolled according to the exclusion criteria (Fig. S1) and randomly assigned to training, validation, and test cohorts at an 8:1:1 ratio. For each patient, a simulation CT was acquired on a Philips Brilliance Big Bore scanner (Philips Healthcare, Best, The Netherlands) with a slice thickness of 5 mm. Target volumes and OARs were contoured by radiation oncologists in accordance with Radiation Therapy Oncology Group guidelines using AccuContour (v3.2; Manteia Technologies Co., Ltd.). All plans were prescribed 45 Gy in 25 fractions and were generated under the institutional planning protocol by medical physicists, with target coverage ensured and OAR sparing prioritized. Plan optimization and final dose calculation were performed in Monaco (clinical version 6.2.2; Elekta AB, Stockholm, Sweden) TPS using a 3-mm dose calculation grid. The planning objectives and dose constraints for targets and OARs are summarized in Table S1.

### Construction of beam band masks

**Fig. 1:**
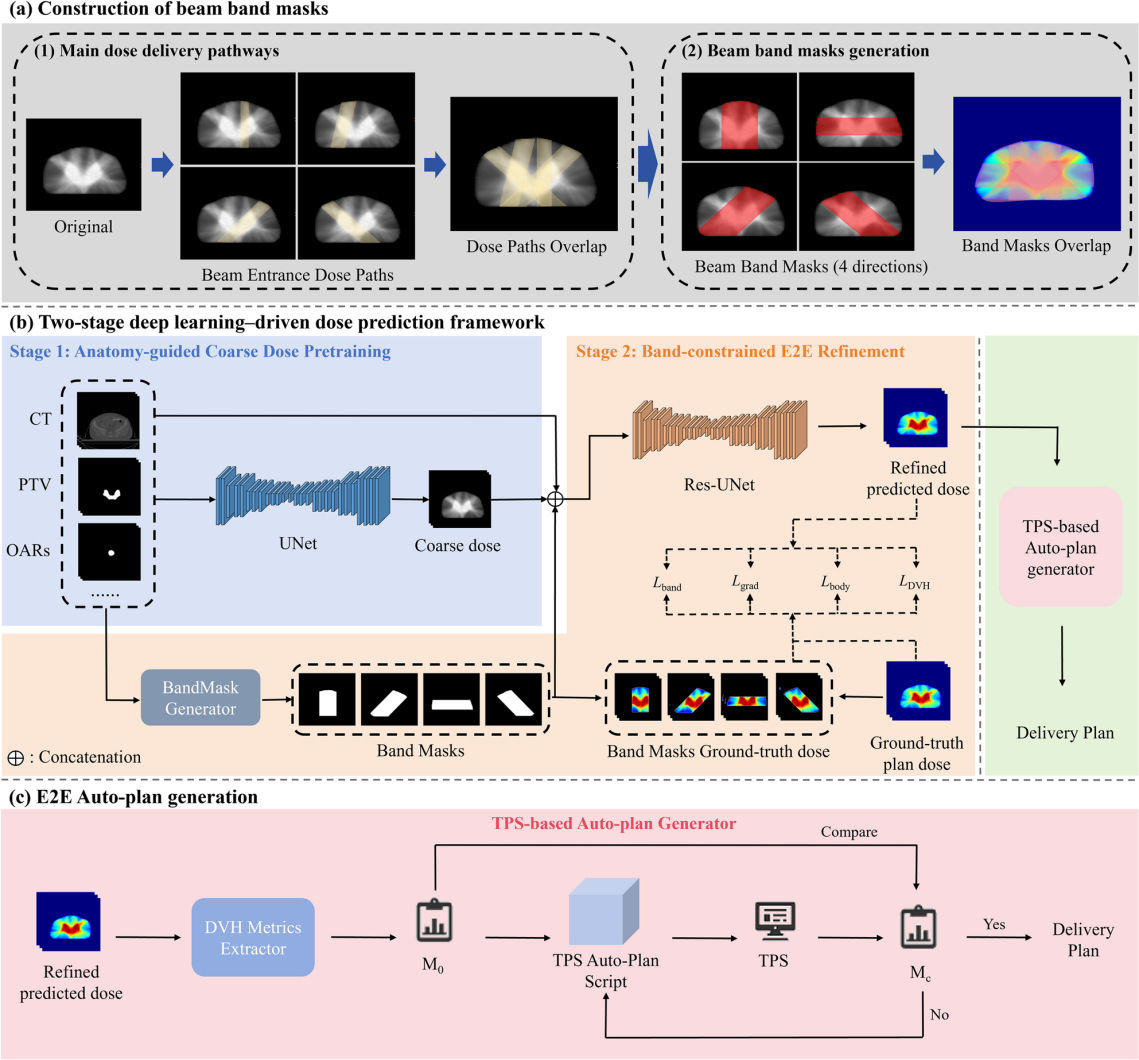
Overview of the proposed E2E VMAT auto-planning workflow. (**a**) Beam band mask generation. Four directional beam band masks (0°, 45°, 90°, and 135°) were constructed to approximate VMAT entrance directionality. (**b**) Two-stage dose prediction framework. A Stage-1 network predicts a coarse dose from CT and structure masks, followed by a Stage-2 refinement network conditioned on beam band masks and optimized with a composite loss. (**c**) TPS-based auto-planning. DVH endpoints from the refined dose define patient-specific optimization targets, which are converted via an in-house Monaco scripting module into executable objectives and iteratively optimized to generate a deliverable plan

An analysis of full-arc VMAT plans for CC patients revealed that the vast majority of dose delivery pathways were predominantly concentrated along several beam-entry directions (Fig. 1a(1)). This directional concentration may reflect inverse optimization mechanism, where OARs dose constraints encouraged beam delivery to avoid traversing critical organs or, when avoidance is not feasible, to lower the beam field weight from those directions. Consequently, four directional beam-band masks were generated on each CT slice. Each beam band mask consisted of two parallel boundaries that are tangent to the plan target volume (PTV) on the respective slice (Fig. 1a(2)). These masks were used as auxiliary inputs to the Stage 2 refinement network.

### Dose prediction model architecture

Before DL network training, CT images must be preprocessed to standardize input dimensions (Appendix A). We developed a cascaded DL framework for E2E prediction of patient-specific 3D dose distributions, and the workflow is shown in Fig. 1b. The framework comprises two consecutive stages. In Stage 1, a 3D U-Net was trained using the planning CT together with binary structure masks of the PTV, selected OARs, and the external body contour as multi-channel inputs [23]. The network outputs a coarse 3D dose map and is supervised against the reference dose using a mean absolute error (MAE) loss. This stage captured the global dose morphology from patient anatomy and structure masks.

The second stage introduces a refinement step conditioned on directional beam band information. Specifically, four directional beam band masks were generated to approximate predominant entrance paths in clinical VMAT plans and used as auxiliary geometric priors. Subsequently, the coarse dose from Stage 1 was concatenated with the CT, structure masks, and the four band masks to form 15 input channels, and a 3D ResU-Net was trained to produce the refined predicted dose [24]. The stage 2 was supervised using a composite objective comprising four loss functions, including *L*_*body*_, *L*_*band*_, *L*_*grad*_ and *L*_*DVH*_, which jointly encourage global accuracy, band-region fidelity, gradient consistency, and DVH-related agreement. Full definitions of each loss function were provided in Appendix B. The second-stage overall loss was defined as a weighted sum:

1 $$\eqalign{ {L_{total}} & = {\lambda _{body}}{L_{body}} + {\lambda _{band}}{L_{band}} \cr & + {\lambda _{grad}}{L_{grad}} + {\lambda _{DVH}}{L_{DVH}} \cr}$$

Detailed network architecture and the training environment and strategy were provided in Appendix C. The full code used in this work is openly available at GitHub (https://github.com/Rick-Ds/Two_stage_E2E_DosePrediction).

### Auto-plan generation

To evaluate whether the refined predicted dose could be translated into deliverable treatment plans of comparable quality to the clinical reference, we performed a TPS script–based auto-planning study, with the workflow shown in Fig. 1c. All procedures were implemented in the Monaco TPS. An in-house script first configured the beam geometry and initialized an automated plan. DVH endpoints were extracted from the refined predicted dose for each region of interest (ROI) and defined as the expected target DVH metrics (*M*_*0*_). Based on *M*_*0*_, plan-specific optimization objectives and constraints were assembled and converted into TPS-compatible parameters to drive inverse optimization. After each optimization finished, DVH metrics of the current plan (*M*_*c*_) were compared with the targets *M*_*0*_. If unmet, only the corresponding objectives were updated for the next iteration: PTV or OARs dose constraint values were increased or decreased by 2%, followed by re-optimization. We limited the maximum number of iterations to no more than five. If the objective cannot be achieved, the TPS scripting module retains an iteration result closest to the goal. A summary of the constraint functions was provided in Table S2.

### Evaluation and statistical analysis

To demonstrate the superiority of the proposed E2E cascaded DL framework for dose prediction, we benchmarked it against top-performing OpenKBP models and subsequent state-of-the-art architectures, including VNet, C3D, DoseNet, HD-UNet, Attention-aware 3D U-Net, and TransDose [12, 25–29]. For fairness, all methods were trained and evaluated on the same dataset with an identical training protocol. Performance was quantified using the official OpenKBP Dose score and DVH score, together with a custom scale-normalized DVH score (snDVH score) [30]. Detailed definitions were provided in Appendix D. Beyond dose prediction, we compared auto-plans with the corresponding clinical reference plans using dose distributions and DVH endpoints. Deliverability was evaluated via linac-based verification of the final auto-plans, and quality assurance results were recorded. To compare our method with reference approaches, paired sample t-tests were performed in SPSS (version 22.0). Statistical significance was defined as a two-tailed *p* value < 0.05.

### Ablation experiments

To examine the interpretability and practical impact of the chosen number of beam bands, we generated band masks with different angular counts and performed dedicated ablation analyses. In addition, to assess the contribution of each loss component, we used the cascaded network as the baseline framework and conducted a series of ablation experiments in which key terms were added sequentially: (1) the global dose consistency loss *L*_*body*_; (2) the band-focused dose loss *L*_*band*_; (3) the band-gradient loss *L*_*grad*_; and (4) the DVH-based numerical loss *L*_*DVH*_.​.

## Results

In the beam-band ablation, a distinct elbow was observed at k = 4. Dose coverage within band-overlap regions increased from 0.39 at k = 1 to 0.95 at k = 4, whereas further increases in k produced only marginal gains of about 0.01 per additional band. Peak GPU memory increased approximately linearly with k, and k = 4 was therefore adopted as a practical trade-off for subsequent experiments (Supplementary Fig. S2).

**Fig. 2:**
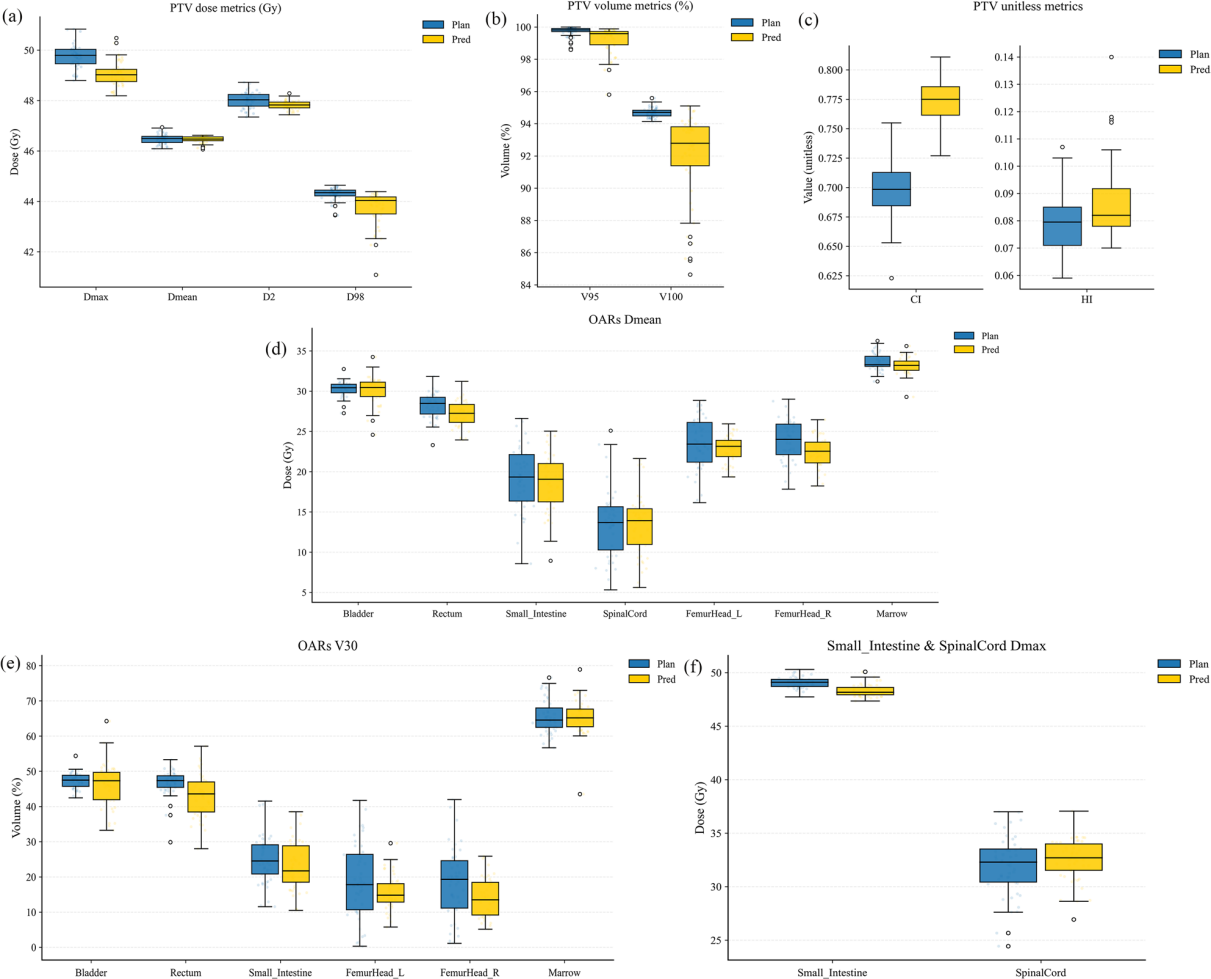
Box plot of critical DVH metrics between the refined predicted dose (yellow) and the clinical plan dose (blue) across PTV and OARs

Benchmarking against six representative DL dose prediction models (Table 1), the proposed method achieved the best overall performance across Dose score, DVH score, and snDVH score, reaching 2.114 ± 0.218 Gy, 1.194 ± 0.295 Gy, and 2.027 ± 0.586, respectively. In addition, we assessed the incremental contribution of the four loss functions. As the key components were introduced sequentially, all three metrics showed an overall downward trend in Table 2; with the final composite loss, improvements were statistically significant across metrics except that the change in Dose score before versus after adding *L*_*DVH*_ did not reach significance. Notably, adding *L*_*band*_ and *L*_*grad*_ reduced the Dose score from 2.407 to 2.176, indicating improved global and band-focused accuracy. After *L*_*DVH*_ was incorporated, DVH score and snDVH score were further reduced by approximately 22% (from 1.526 to 1.194) and 18% (from 2.475 to 2.027), respectively, suggesting that the DVH-based constraint improved ROI-relevant endpoint agreement. In contrast, the change in Dose score after adding *L*_*DVH*_ (2.176 to 2.114) was modest and did not show a clear additional gain. Figure 2 further illustrates the prediction accuracy of the proposed method for DVH metrics across the relevant ROIs. Overall, the predicted and reference dose distributions showed close agreement for PTV indices including D_max_, D_mean_, D_2%_, D_98%_, V_95%_ and HI, and for OAR-related endpoints including D_mean_, V_30_, and the Dmax of the small intestine and spinal cord.

**Table 1:** Quantitative comparison between SOTA methods and our method in terms of Dose score, DVH score and snDVH Score

Model	Dose Score [Gy]	DVH Score [Gy]	snDVH Score
VNet	2.336 ± 0.335*	1.917 ± 0.426*	3.068 ± 0.748*
C3D	2.319 ± 0.259*	1.906 ± 0.400*	2.794 ± 0.663*
DoseUNet	2.227 ± 0.280*	1.866 ± 0.364*	2.646 ± 0.672*
HD UNet	2.204 ± 0.283*	1.651 ± 0.333*	2.535 ± 0.703*
Attention-aware 3D UNet	2.180 ± 0.337	1.565 ± 0.508*	2.582 ± 0.641*
TransDose	2.136 ± 0.211	1.395 ± 0.293*	2.424 ± 0.666*
**Proposed**	**2.114 ± 0.218**	**1.194 ± 0.295**	**2.027 ± 0.586**

*: *P* value < 0.05 versus the proposed model by two-sided paired t-test;

**Table 2:** Experimental results of ablation studies on our proposed method

Method	Dose Score [Gy]	DVH Score [Gy]	snDVH Score
Baseline	2.407 ± 0.230*	1.983 ± 0.337*	3.037 ± 0.595*
Baseline + *L*_*band*_	2.245 ± 0.262*	1.641 ± 0.331*	2.763 ± 0.562*
Baseline + *L*_*band*_ + *L*_*grad*_	2.176 ± 0.290	1.526 ± 0.362*	2.475 ± 0.653*
Baseline + *L*_*band*_ + *L*_*grad*_ + *L*_*DVH*_	2.114 ± 0.218	1.194 ± 0.295	2.027 ± 0.586

*: *P* value < 0.05

*P*value: calculated by Paired t-test

**Fig. 3:**
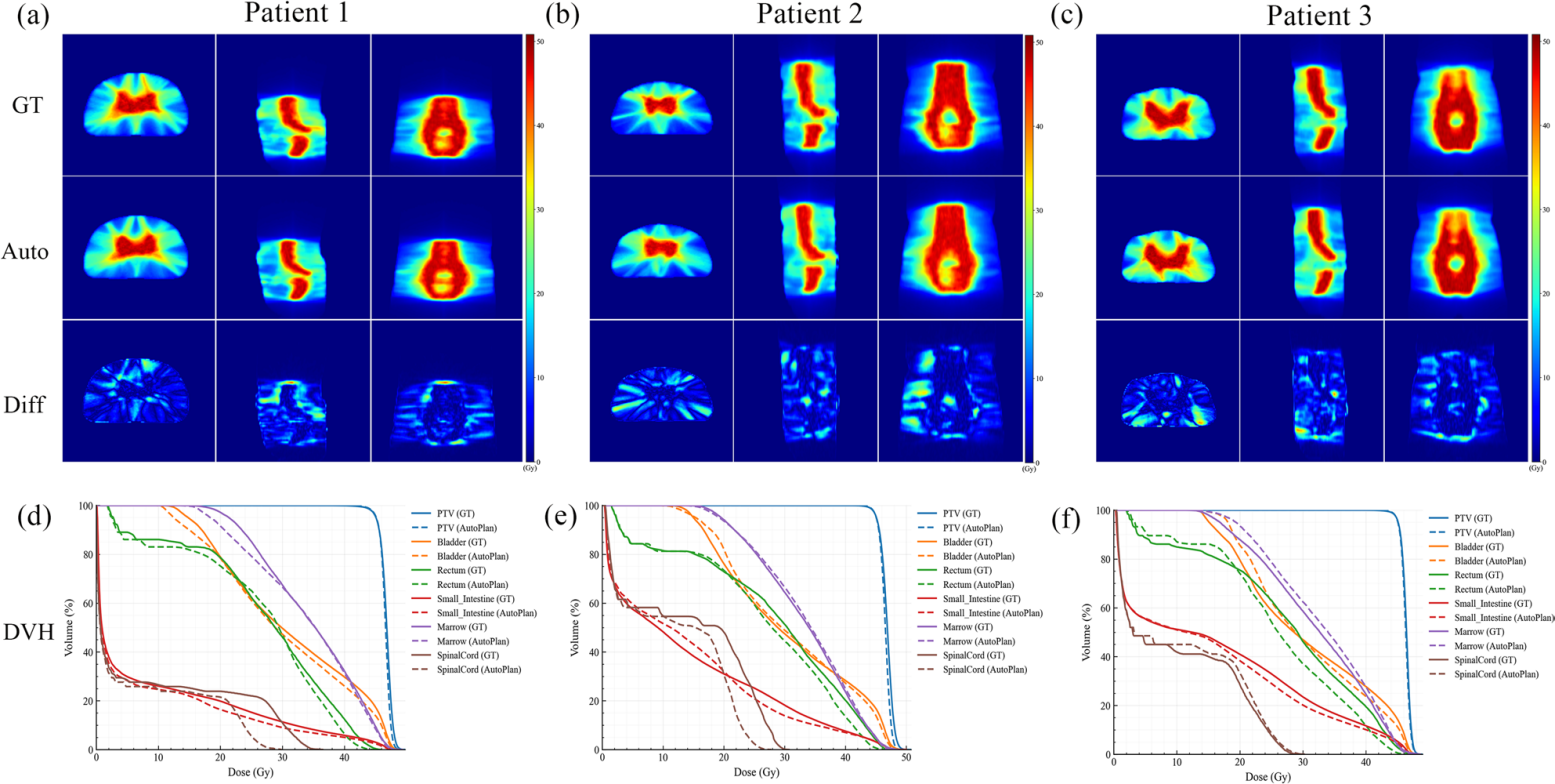
Representative case comparisons between the clinical reference plan and the E2E auto-plan. (**a**)-(**c**) For three cases, axial/coronal/sagittal dose distributions are shown for the ground-truth clinical plan (GT), the TPS-generated auto-plan (Auto), and the voxel-wise difference map (Diff; Auto-GT). (**d**)-(**f**) Corresponding DVH curves for PTV and OARs. Solid lines denote GT and dashed lines denote Auto

The quantitative evaluation of the clinical reference plans and the E2E auto-plans were presented in Table 3. Target coverage was comparable, with no significant differences in PTV V_95%_ (*p* = 0.195) or V_100%_ (*p* = 0.650), indicating that clinical coverage requirements were maintained. Small but significant reductions were observed in PTV D_2%_ (*p* = 0.001) and HI (*p* = 0.002), and they reflected a reduction in high-dose regions of target and an improvement in dose homogeneity. For OARs, there was no statistically significant difference in marrow DVH metrics between the two plan types, with similar D_mean_ and V_30Gy_ (*p* = 0.714 and *p* = 0.213). Notably, all DVH metrics for the bladder, rectum, small intestine, and spinal cord were significantly reduced in the E2E auto-plans, with relative decreases of 2% – 35% compared with the reference plans (all *p* < 0.05), whereas both femoral heads received higher doses, with an approximately 1 Gy increase in D_mean_ and a 4% – 6% increase in V_30Gy_ compared with the reference plans. Qualitative examples were shown in Fig. 3. Across three representative cases, the high-dose regions were largely consistent between the E2E auto-plans and reference plans, while improved conformity was observed in the 20 – 30 Gy dose range. The corresponding DVHs showed nearly overlapping PTV coverage, accompanied by systematic shifts of the bladder, rectum, small bowel, and spinal cord curves toward lower doses within clinically relevant ranges, indicating improved OAR sparing without compromising target coverage, highlighting the dosimetric advantages of the E2E auto-planning approach.

**Table 3:** Quantitative evaluation of DVH metrics of PTV and OARs of GT plan and E2E auto-plan

Structures	DVH metrics	GT plan	E2E auto-plan	*P*-value
PTV	Dmax(Gy)	49.74 ± 0.47	49.59 ± 0.49	0.088
	Dmean(Gy)	46.48 ± 0.20	46.34 ± 0.19	0.000
	V95(%)	99.70 ± 0.30	99.80 ± 0.20	0.195
	V100(%)	94.70 ± 0.30	94.70 ± 0.30	0.650
	D98%(Gy)	44.29 ± 0.26	44.35 ± 0.21	0.062
	D2%(Gy)	48.00 ± 0.32	47.82 ± 0.32	0.001
	CI	0.70 ± 0.03	0.70 ± 0.03	0.070
	HI	0.08 ± 0.01	0.07 ± 0.01	0.002
Bladder	Dmean(Gy)	30.25 ± 0.97	29.58 ± 1.70	0.014
	V30(%)	47.30 ± 2.30	44.80 ± 5.70	0.002
	V40(%)	28.90 ± 2.80	24.90 ± 5.20	0.000
	V45(%)	16.80 ± 3.10	13.60 ± 3.70	0.000
Rectum	Dmean(Gy)	28.20 ± 1.54	26.81 ± 1.90	0.000
	V30(%)	46.70 ± 3.80	40.40 ± 6.70	0.000
	V40(%)	20.70 ± 3.80	13.40 ± 4.30	0.000
	V45(%)	5.20 ± 3.00	2.00 ± 2.20	0.000
Small_Intestine	Dmax(Gy)	49.08 ± 0.51	48.77 ± 0.54	0.005
	Dmean(Gy)	19.12 ± 3.95	18.10 ± 3.89	0.000
	V30(%)	25.10 ± 6.90	21.00 ± 7.00	0.000
SpinalCord	Dmax(Gy)	31.98 ± 2.81	29.53 ± 2.61	0.000
	Dmean(Gy)	13.53 ± 4.16	12.34 ± 3.82	0.000
Marrow	Dmean(Gy)	33.63 ± 1.07	33.56 ± 1.34	0.714
	V30(%)	65.50 ± 4.80	64.30 ± 6.10	0.213
Left Femoral Head	Dmean(Gy)	23.49 ± 3.25	24.50 ± 2.93	0.010
	V30(%)	18.90 ± 10.50	24.40 ± 10.80	0.001
	V40(%)	0.40 ± 0.70	1.00 ± 1.30	0.002
Right Femoral Head	Dmean(Gy)	23.86 ± 2.57	25.05 ± 2.21	0.006
	V30(%)	18.40 ± 9.90	22.90 ± 8.90	0.004
	V40(%)	0.60 ± 0.90	1.00 ± 1.30	0.014

P-value: calculated by Paired t-test; P value < 0.05 was considered statistically significant

Deliverability verification using gamma analysis with a 3%/3 mm criterion yielded comparable passing rates: the E2E auto-plans achieved a mean of 98.1% (range: from 96.7% to 99.0%), while the reference plans achieved 97.9% (range: from 96.8% to 99.3%) in table S3, indicating similar deliverability to clinical plans.

## Discussion

We developed and validated a closed-loop VMAT auto-planning workflow that converts patient-specific anatomy into deliverable plans. Target coverage was preserved while DVH metrics for key OARs improved, and deliverability was supported by delivery verification. Dose prediction was conditioned on interpretable, direction-aware beam band priors and trained with a composite loss function to better capture steep dose fall-off and target-OAR abutment interfaces. Predicted dose was translated, via TPS script module, into Monaco-executable individualized objectives and iteratively optimized, enabling robust transfer from prediction to actionable constraints and reducing repeated manual tuning, thereby providing a practical route for integrating prediction into E2E clinical VMAT planning.

Unlike other DL medical image regression tasks such as auto-segmentation and image reconstruction, where inputs are often relatively direct, dose prediction typically requires deliberate input design [31]. This is because plan generation is shaped not only by anatomy, but also by clinical preferences and physical realities such as beam arrangement and energy deposition constraints [32, 33]. Sun et al. proposed a physics voxel-based optimization strategy and reported mean-dose reductions of 3.1, 6.2 and 4.5 Gy in the bladder and bilateral femoral heads compared with manual plans [34]. In the work by Teng et al., fixed-field characteristics specific to IMRT were leveraged: they simulated ray paths according to PTV position and beam angles to construct beam masks as network inputs, and demonstrated reduced prediction errors across individual beam angles [13]. Xiong et al. designed normalized distance-aware beam plates and mass density maps as physics-informed priors, and their ablation experiments showed a 5.8% reduction in MAE after incorporating such priors [35]. Considering the dynamic modulation and continuous gantry rotation intrinsic in VMAT, we constructed four directional beam band masks to approximate the directionality of dose incidence and used them as geometric priors as network inputs. In ablation experiments, adding the band masks reduced the Dose score from 2.407 to 2.245 (a relative decrease of 6.7%), and the full configuration further reduced it to 2.176 (a relative decrease of 9.6%). These results indicate that explicitly encoding direction-aware band priors in the model input yields a consistent improvement in dose prediction performance.

Table 1 showed that the proposed two-stage cascaded dose prediction framework outperformed the other evaluated models. A key reason was that many prior DL approaches, despite careful architectural design, were still trained primarily with MAE or other similar mean error-related objectives. Such objectives encourage learning a mapping towards the conditional mean or median, which can produce overly smooth dose distributions and attenuate boundary details [36, 37]. This is undesirable when high-gradient transitions are clinically critical. Nguyen et al. explored differentiable DVH-based and adversarially inspired losses, demonstrating their utility for training dose models and generating Pareto-optimal radiotherapy dose distributions [38]. In our framework, MAE was retained in the first stage to learn the global dose distribution, while the second stage introduces a composite objective (*L*_*band*_ + *L*_*grad*_ + *L*_*DVH*_) to refine band-region behaviour, local gradient transitions, and DVH consistency. This design aims to improve prediction accuracy while preserving global coherence. From a task-decomposition perspective, the cascade allows the model to learn the low-frequency/global dose pattern first and then correct high-frequency/local discrepancies, which can reduce the learning difficulty of the refinement stage and improve training robustness. Teng et al. adopted a related strategy by decomposing a global coarse dose into multiple field doses, refining them on a per-field basis, and then aggregating to obtain the final 3D dose distribution, achieving improved predictive performance [13]. In addition, our cohort was comparatively large, and organ-wise dose distributions span a broad range (Supplementary Fig. S3), which provided richer supervision for the refinement stage during error correction.

Several studies had explored how to translate predicted dose into actionable planning. Shen et al. discretized predicted dose in specific regions to construct optimization objectives and used a two-step optimization scheme [20]. Choi et al. automatically extracted structure-specific objective values and weights from predicted dose and wrote them into a TPS to generate plans [39]. Church et al. combined dose mimicking with a residual U-Net to predict deliverability-related elements and converted them into DICOM-RT plans for evaluation [40]. Relative to these studies, the present work emphasized three aspects. First, it targets the VMAT setting, where dynamic modulation and continuous rotation impose directionality and high-gradient characteristics that were not well represented by anatomy alone. Second, the predicted dose was transformed through TPS scripting into Monaco-executable individualized constraints and iteratively optimized to produce the final plan, rather than remaining solely at the level of a predicted distribution. Third, we incorporated gamma analysis to support plan deliverability. Moreover, Table 3 indicated that, with target coverage maintained, DVH endpoints for most key OARs decreased overall. Notably, Dmean and V30 for the bilateral femoral heads increased by approximately 1 Gy and 5%, respectively. This might reflect a redistribution of dose contributions across directions during optimization. Additionally, femoral heads were relatively small structures and their DVH metrics could be more sensitive to localized dose variations. Importantly, femoral head doses in the E2E auto-plans still remained within clinically acceptable ranges. In future work, we would further refine the optimization strategy for small-volume bony structures such as the femoral heads, introducing additional constraints and/or objective rebalancing to prevent metric drift and ideally achieve neutral or reduced values.

This study had several limitations. First, the diversity of the cohort enrolling and prescription choices remains limited. Therefore, generalizability across different prescriptions, especially concurrent boost therapy with additional high-dose subvolumes and different dose gradients, as well as broader patient populations, requires further validation. In the further study, We plan to expand the sample size and include additional prescription schemes in CC cohorts. Second, plan quality is partly contingent on the accuracy of dose prediction; we will continue to improve the predictive network and loss design to enhance robustness in steep-gradient and target-OAR adjacent regions. Third, although we achieved E2E auto-planning within the Monaco TPS, cross-TPS portability has not been established and will require dedicated interface development and consistency assessments. Finally, our experiments focused on a single tumor site; validation across multiple tumor sites is essential. A multi-site auto-planning framework, adapted to varying clinical requirements, is expected to offer broader applicability and improved robustness, and this will facilitate more reliable integration of the proposed workflow into clinical radiotherapy practice.

## Conclusion

We propose a closed-loop framework that converts anatomy into deliverable VMAT plans by integrating direction-aware beam-band priors, DVH-guided learning, and Monaco TPS scripting for optimization. The method preserves target coverage, improves OARs’ DVH endpoints, and demonstrates deliverability with gamma verification. This approach reduces manual trial-and-error and generates deliverable plans, which has the potential to substantially improve the efficiency and consistency of CC radiotherapy planning, with promising prospects for rapid auto-plan generation in routine practice.

## Supplementary Information

Below is the link to the electronic supplementary material.

Supplementary Material 1

## Data Availability

The datasets used and/or analyzed during the current study are available from the corresponding author on reasonable request.

## Abbreviations

IMRT: Intensity-modulated radiotherapy
VMAT: Volumetric modulated arc therapy
CC: Cervical cancer
OARs: Organs at risk
ATP: Automated treatment planning
KBP: Knowledge-based planning
DVH: Dose-volume histogram
3D: Three-dimensional
DL: Deep learning
TPS: Treatment planning system
E2E: End-to-end
PTV: Plan target volume
MAE: Mean absolute error
ROI: Region of interest
snDVH score: Scale-normalized DVH score
