# Supplementary material for: A deep learning-driven automated treatment planning framework for cervical cancer patients treated with volumetric modulated arc therapy
Source: Radiat Oncol. 2026 Apr 14;21:75. doi: 10.1186/s13014-026-02842-9 (PMC13173708; doi:10.1186/s13014-026-02842-9)
Supplement: Supplementary file 1 — Supplementary Material 1 [file 13014_2026_2842_MOESM1_ESM.docx]

**Supplementary Materials**


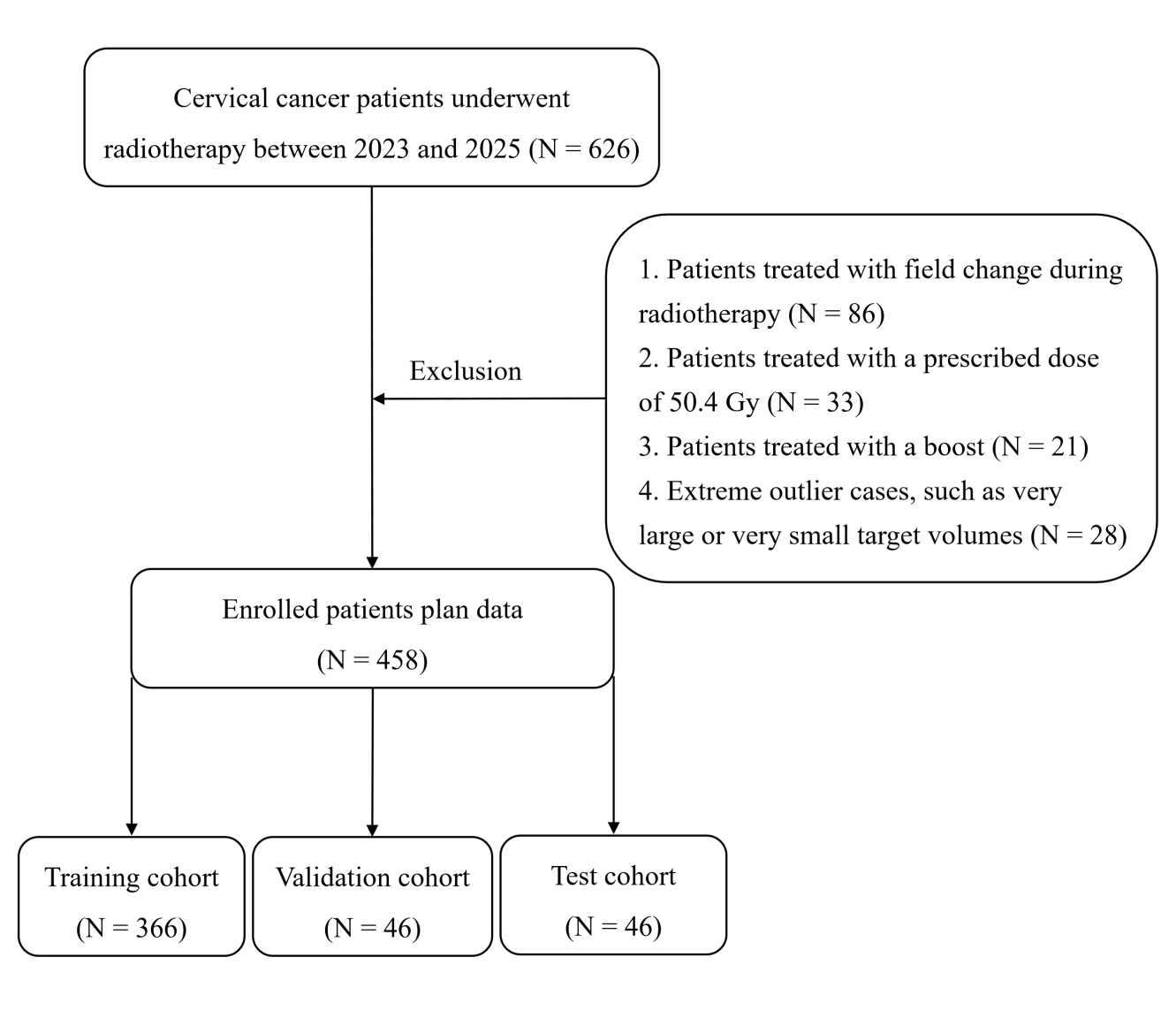


**Supplementary Figure S1.** Exclusion criteria of patients

| **Supplementary Table S1. Overview of the local clinical dose criteria.** | | | |
| --- | --- | --- | --- |
| Structure | Dosimetric parameter | Per Protocol | Variation Acceptable |
| **Target** |  |  |  |
| PTV | V_4500cGy_ [%] | ≥ 95 | ≥ 93-95 |
|  | D_0.03cc_ [cGy] | < 4950 | < 4950-5150 |
| **OARs** |  |  |  |
| Bladder | V_4000cGy_ [%] | < 40 | < 40-43 |
|  | D_0.03cc_ [cGy] | < 4800 | < 4800-4950 |
| Rectum | V_4000cGy_ [%] | < 40 | < 40-45 |
|  | D_0.03cc_ [cGy] | < 4800 | < 4800-4950 |
| Small Intestine | V_4000cGy_ [%] | < 30 | < 30-40 |
|  | D_max_ [cGy] | < 4800 | < 4800-5150 |
| Spinal Cord | D_max_ [cGy] | < 4000 | < 4000-4500 |
| Marrow | D_mean_ [cGy] | < 2700 | < 2700-2900 |
|  | V_2000cGy_ [%] | < 66 | < 66-75 |
| Femur Head L/R | V_4000cGy_ [%] | < 5 | < 5-8 |

**Appendix A: Imaging preprocessing**

Firstly, all images were exported from the TPS at the native resolution of 512 × 512 × 64, including the CT and dose distribution. Using in-house code, spatial coordinates were extracted from the RT Structure file to generate ROI-specific masks. Beam band masks were then derived from the PTV mask, and all masks were created to match the CT in both image dimensions and affine transformation. To improve computational efficiency and facilitate learning of dose continuity, all volumes were resampled to 256 × 256 × 64 for training and inference: CT and dose volumes were downsampled using trilinear interpolation, whereas all masks were downsampled using nearest-neighbor interpolation. Predictions were subsequently mapped back to the native dose grid using trilinear interpolation.

**Appendix B: Mathematical definitions of *L*_body_, *L*_band_, *L*_grad_ and*L*_DVH_ in stage two**

Let $\hat{\text{d}}$ and $\text{d}$ denote the predicted and reference 3D dose grids, respectively, normalized to [0, 1]. The physical dose in Gy is obtained as $\hat{D}=D_{\text{max}}\cdot\hat{d}$ and $D=D_{\text{max}}\cdot\text{d}$, where $D_{\text{max}}$ is the normalization constant ($D_{\text{max}}$ = 52 Gy in our implementation).

A voxel-wise mean absolute error (MAE) was applied over the full 3D grid:

(1)

where $\text{n}$ indexes voxels and $N=DHW$ (or the number of voxels included by the training crop).

Let $\left\{\text{m}^{(k)}\right\}_{k=1}^{K}$ be $K$ band/strip masks. The band loss aggregates absolute errors only within the band regions and normalizes by the total number of band voxels (implemented per patient, then averaged over valid patients):

(2)

where B denotes patients in the batch with at least one band voxel (denominator > 0).

We enforced spatial dose-gradient consistency using a 3D gradient L1 loss computed via forward differences along $\text{z}$, $\text{y}$, and $\text{x}$:

$\Delta_{\text{z}}\text{d}\left(i\right)=d\left(i+e_{z}\right)-d\left(i\right),\Delta_{\text{y}}\text{d}\left(i\right)=d\left(i+e_{y}\right)-d\left(i\right),\Delta_{\text{x}}\text{d}\left(i\right)=d\left(i+e_{x}\right)-d\left(i\right).$ (3)

The loss is:

(4)

where denotes the mean absolute value over the corresponding valid voxel set. If a region mask m is provided, only voxel pairs fully inside the mask are included, e.g., $\text{Ω}_{\text{z}}=\left\{i:m\left(i\right)m\left(i-e_{z}\right)=1\right\}$; otherwise, the mean is taken over all forward-difference voxels in each direction.

For each OAR $\text{r}$ with mask $M_{\text{r}}$, we penalized discrepancies of DVH-relevant endpoints. First, mean dose (Gy) is:

$D_{\text{mean}}\left(d,r\right)=D_{\max}\cdot\frac{\sum_{n} M_{r,n}d_{n}}{\sum_{n} M_{r,n}}.$ (5)

A squared error on mean dose is accumulated:

(6)

If enabled for an organ, the predicted maximum dose is approximated by a log-sum-exp “soft-max” (Gy):

(7)

while the reference $D_{\text{max}}\left(d,r\right)$ is computed with a hard threshold. The squared error $\text{l}_{V_{x}}\left(r\right)=\left(V_{x}^{soft}-V_{x}\right)^{2}$ is accumulated over all specified $\text{x}$.

The DVH loss sums all applicable terms across organs and applies a batch mean:

$L_{DVH}=\frac{1}{B}\sum_{\text{b}=1}^{B} \sum_{r\in\Re} \left(l_{mean}^{(b)}\left(r\right)+\prod_{\max} \left(r\right)l_{\max}^{(b)}\left(r\right)+\sum_{x\in\aleph_{\text{r}}} l_{V_{x}}^{(b)}\left(r\right)\right),$ (8)

where $\Re$ is the set of OARs with defined constraints, and $\aleph_{\text{r}}$ is the set of $V_{\text{x}}$ thresholds for organ $\text{r}$.

**Appendix C: Deep learning network architecture and details**

The proposed framework was implemented as a cascaded two-stage predictor in which the output of Stage 1 is fed into Stage 2, and both stages are executed sequentially within a single forward pass. The implementation explicitly concatenates the Stage-1 coarse dose into the Stage-2 input so that the refinement network learns to correct the coarse prediction using additional priors.

**Input composition and channel usage.** The model uses the planning CT, binary structure masks, and directional band masks as inputs. Stage 1 takes the CT together with nine binary masks (PTV, selected OARs, and the external body contour), where each mask is provided as an independent channel. Stage 2 uses the same CT and structure masks, concatenates the Stage-1 coarse dose as an additional channel, and further appends four directional band-mask channels. In the code, the multi-channel tensor is explicitly split so that Stage 1 uses CT + structure masks, while Stage 2 uses CT + structure masks + coarse dose + band masks.

**Stage 1 network (3D U-Net).** Stage 1 follows a standard 3D U-Net with four encoder levels and four decoder levels. Each encoder level applies a two-convolution block consisting of two 3×3×3 convolutions, each followed by batch normalization and ReLU activation. Downsampling is performed by 2 × max pooling between encoder levels. The decoder mirrors the encoder using transposed 3D convolutions with kernel size 2 and stride 2 for upsampling, followed by concatenation with the corresponding encoder feature map (skip connection) and another two-convolution block of the same form. The final prediction layer is a 1 × 1 × 1 convolution, and the output activation is linear (identity), consistent with dose regression. In the training configuration used in this work, the Stage-1 U-Net was instantiated with a base number of filters of 8, and the feature widths follow the standard doubling pattern across depth. The implementation performs four 2 × pooling operations; therefore, input dimensions are recommended to be divisible by 16 to avoid shape mismatches in the encoder–decoder pathway.

**Stage 2 network (3D ResU-Net).** Stage 2 is a 3D residual U-Net variant built from residual blocks. Each residual block contains two 3 × 3 × 3 convolutions with batch normalization, with ReLU applied after the first convolution and again after adding the residual connection. When the number of input and output channels differs, a 1 × 1 × 1 convolution with batch normalization is used as a projection on the skip branch to match dimensions. The encoder uses residual blocks with 2 × max pooling between levels, and the decoder upsamples using transposed 3D convolutions (kernel size 2, stride 2), concatenates skip features from the encoder, and applies residual blocks for refinement. The final layer is a 1 × 1 × 1 convolution with linear (identity) activation to output the refined dose. In this study, the Stage-2 ResU-Net was instantiated with a base number of filters of 16, following the same doubling pattern across depth.

**Training environment and strategy.** All deep-learning models were implemented in Python 3.7 using PyTorch 1.9 and trained on two NVIDIA GeForce RTX 3090 GPUs (24 GB). We adopted an iteration-wise warm-up followed by cosine annealing for the learning-rate schedule. The batch size was set to 2. Each model was trained for up to 400 epochs, and the best-performing checkpoint was selected using early stopping with a patience of 50 epochs.

| **Supplementary Table S2. VMAT constraint functions of auto-plan in Monaco.** | | |
| --- | --- | --- |
| Structure | Cost Function | Reference Dose (Gy) |
| **Target** |  |  |
| PTV | Target Penalty |  |
|  | Quadratic Overdose | 4600 |
| **OARs** |  |  |
| Bladder | Overdose DVH | 3000 |
|  | Overdose DVH | 4000 |
| Rectum | Maximum Dose |  |
|  | Overdose DVH | 3000 |
|  | Overdose DVH | 4000 |
| Small Intestine | Maximum Dose |  |
|  | Overdose DVH | 3000 |
| Spinal Cord | Maximum Dose |  |
| Marrow | Overdose DVH | 3000 |
| Femur Head L | Overdose DVH | 4000 |
| Femur Head R | Overdose DVH | 4000 |
| Body | Maximum Dose |  |
|  | Quadratic Overdose | 4000 |
|  | Quadratic Overdose | 3600 |
|  | Quadratic Overdose | 3200 |
|  | Quadratic Overdose | 2900 |
|  | Maximum Dose |  |

**Appendix D: Mathematical definitions of Dose score, DVH score and snDVH score**

**Dose score**. Prediction accuracy at the voxel level was quantified using the mean absolute error (MAE) of the dose distribution within the external body contour. Let $d^{pred}\left( v \right)$ and $d^{gt}\left( v \right)$ denote the predicted and reference dose (Gy) at voxel $v$, and $\Omega_{Body}$ the set of voxels inside the body mask. The dose score was defined as:

**DVH score**. DVH-based agreement was evaluated using an OpenKBP DVH score computed as the mean absolute error across a predefined set of DVH endpoints:

where $M_{k}\left( \cdot\right)$ denotes the k-th DVH endpoint. The set $\mathcal{S}_{KBP}$ comprised PTV D_99_, D_95_ and D_1_, and for each OAR, and D_0.1cc_ and D_mean_ (all in Gy).

**Scale-normalized DVH score (snDVH score)**. To provide a scale-normalized summary of DVH/plan-quality agreement, we computed a scale-normalized DVH score (snDVHScore) as the mean of normalized endpoint errors across a predefined set of targets and OAR metrics. For each endpoint $M_{k}\left( \cdot\right)$ evaluated on the predicted and reference dose distributions, $d^{pred}$ and $d^{gt}$, the normalized error $\Delta_{k}$ was defined as:

The snDVHScore was then obtained by averaging $\Delta_{k}$ over all endpoints:

The endpoint set $\mathcal{S}_{sn}$ included PTV D_mean_, D_98_, D_92_, V_95_, V_100_, conformity index (CI), and homogeneity index (HI); bladder/rectum/femoral heads/marrow D_mean_, V_30_ and V_40_; and small bowel/spinal cord D_max_. Here, $V_{x}$ denotes the fractional organ volume receiving ≥ x Gy. Lower snDVHScore indicates closer agreement with the reference DVH/plan-quality metrics.


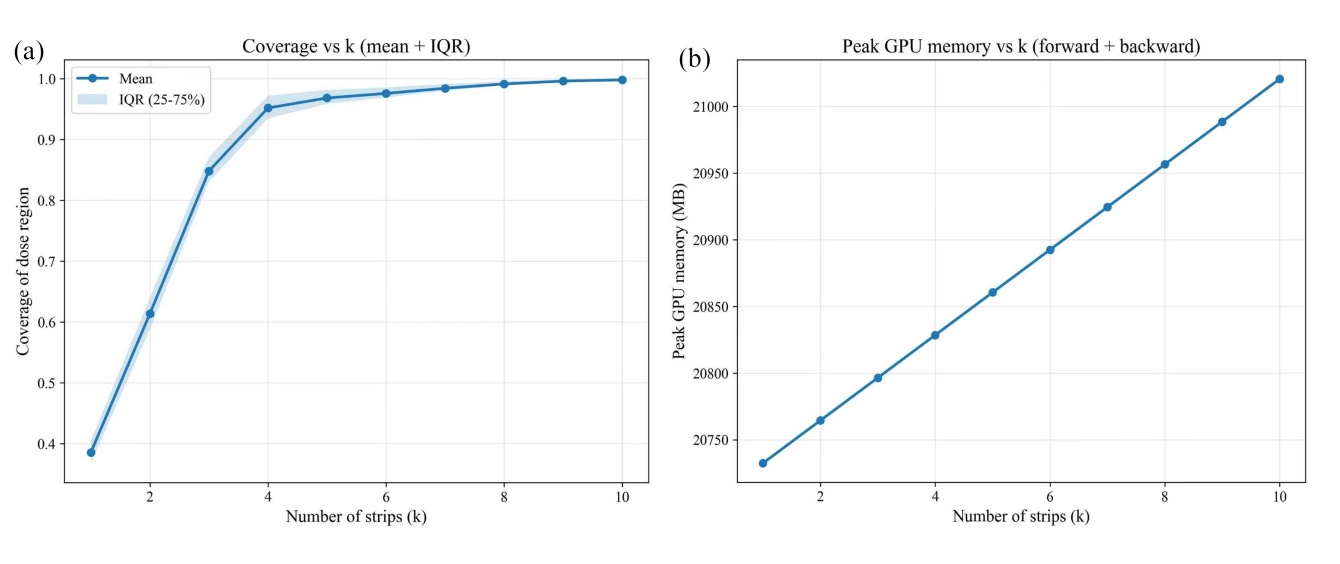


**Supplementary Figure S2.** Impact of the number of strip bands (k) on coverage and computational cost. (a) Coverage of the dose region as a function of k, reported as the cohort mean with interquartile range (IQR, 25–75%). (b) Peak GPU memory consumption during training (forward + backward) versus k.

| **Supplementary Table S3. Gamma passing rate in E2E auto-plans and reference plans.** | | |
| --- | --- | --- |
| Gamma passing rate | E2E auto-plan | Reference plan |
| 3%/3mm (%) | 98.1 ± 0.8 | 97.9± 0.8 |


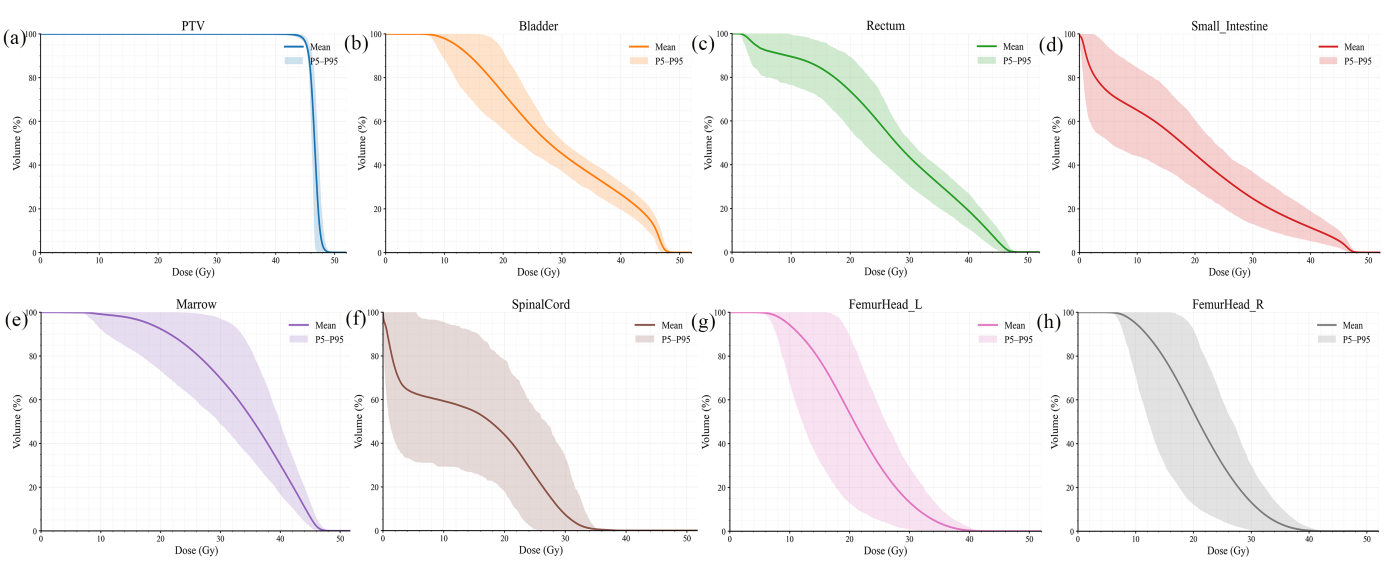


**Supplementary Figure S3.** Cohort-level dose–volume histograms (DVHs) for the planning target volume (PTV) and organs at risk (OARs). Solid lines represent the mean DVH, and shaded bands indicate the 5th–95th percentile range (P5–P95) across patients for (a) PTV, (b) bladder, (c) rectum, (d) small intestine, (e) marrow, (f) spinal cord, (g) left femoral head, and (h) right femoral head.
